# The Spectrum of Clinical Pharmacy Services in a Non-University Hospital—A Comprehensive Characterization Including a Risk Assessment for Drug-Related Problems and Adverse Drug Reactions

**DOI:** 10.3390/pharmacy13060164

**Published:** 2025-11-06

**Authors:** Olaf Zube, Wiebke Schlüter, Johanna Dicken, Jan Hensen, Thilo Bertsche

**Affiliations:** 1 Bundeswehr Hospital Pharmacy, 22049 Hamburg, Germany; 2 Clinical Pharmacy Department, Institute of Pharmacy, Medical Faculty, Leipzig University, 04103 Leipzig, Germany; 3 Drug Safety Center, Medical Faculty, Leipzig University, 04103 Leipzig, Germany

**Keywords:** clinical pharmacy services, hospital pharmacy, pharmacy management, decision management, drug-related problems, adverse drug reactions

## Abstract

*Background:* Clinical pharmacy services (CPS) have been shown to confer significant advantages in patient care. It remains to be clarified how CPS resources are allocated across routine care settings. It remains to be clarified which recommendations are made to resolve the drug-related problems (DRP) identified by CPS and which adverse drug reactions (ADR) actually arise from the identified DRP. *Methods:* Following positive ethical approval, patient chart analyses, evaluation of pharmacy documentation on CPS and pharmacist interviews were performed to characterize CPS at all medical departments of the Bundeswehr Hospital Hamburg. We developed and pre-tested instruments for standardization: A Standard Operating Procedure (SOP) for the practical exercise and documentation of CPS by the pharmacists performing them, a standardized form (checklist) for retrospective data collection as part of this study, and a standardized questionnaire for conducting the pharmacist interviews including a risk assessment according to the NCC-MERP score. *Results:* In total, 1000 CPS were documented in 504 patients (mean age: 69.95 years; 229 female) on 16,705 treatment days. A total of 66.87% CPS was initiated when pharmacists participated in ward rounds. In all CPS, “Indications” was the topic addressed most frequently (37.70%). “Agents for obstructive respiratory diseases” was the most frequently involved drug class (11.32%). The most frequent processing time per CPS was 16–30 min (48.61%). The number of CPS ranged from 0.36/100 treatment days in dermatology to 12.47 in oncology. Severity of 358 DRP was classified “very severe” (5.03%), “severe” (42.74%), “moderate” (34.36%), “low” (15.08%), “very low” (1.40%), or “without impact” (1.40%). The probability of DRP occurrence was classified as “high” in 13.13% and “very high” in 3.35%. In 15.36% of the DRP, an ADR actually occurred. In 504 patients, 932 specific recommendations were forwarded to solve the DRP identified during CPS. Of those, 53.97% were implemented. *Conclusions:* In almost all CPS, a considerable number of DRP with serious clinical consequences were identified. Half of the forwarded recommendations were implemented.

## 1. Introduction

If impaired elimination capacity or drug-drug interactions are not considered in patients taking multiple medications, drug-related problems (DRP) are possible, which can lead to clinical consequences [1]. Vulnerable groups, for example older patients, are particularly affected by these kinds of clinical consequences that can lead to ineffectiveness of the therapy or preventable risks such as adverse drug reactions (ADR) [2]. Particular dosage forms (e.g., inhalative devices), complex therapy regimens (e.g., in oncology), and expensive therapies (e.g., in oncology) are therapy-related variables that can represent additional risk factors for adverse pharmacotherapeutic or pharmacoeconomic outcomes when DRP occur [3].

This results in an increased demand for advice for the treating physicians whenever it is a matter of correctly chosen therapy (pharmacodynamically appropriate) [4], correctly dosed drugs (pharmacokinetically appropriate) [5], correctly administered dosage form (pharmaceutically appropriate) [6], and cost-efficient therapy (pharmacoeconomically appropriate) according to the current evidence [7].

All these factors offer worthwhile starting points for integrating pharmacists into an interprofessional treatment team [8]. Clinical pharmacy services (CPS) have proven to be an effective and efficient strategy in this sense, according to the international literature, even in low-income countries [9]. However, the focus tends to be on particularly vulnerable patient groups such as intensive care patients [10] and university settings [11]. Different medical areas with other patient groups and non-university hospitals have not yet received the same level of attention, even if a considerable fraction of patients would benefit. In addition, most studies do not present meaningful data on the clinical consequences of DRP addressed via CPS, such as actually occurring events predicted by the identified DRP (i.e., ADR).

In this study, we therefore characterized the comprehensive CPS offered to the full spectrum of a non-university hospital. We aimed to assess the following research questions in this study: What is the number of CPS per patient/treatment day? How many patients are involved in CPS per department? How are CPS requests processed? What topics are addressed in the CPS? Which drug groups are addressed in the CPS? How much time is required for the CPS? How are the potential severity of DRP and the number of actually occurring ADR during CPS assessed? How likely is the occurrence of the DRP identified in the CPS? What recommendations are given to resolve the DRP identified in the CPS? What is the acceptance of the recommendations identified in the CPS for resolving the DRP?

## 2. Materials and Methods

*Patients and setting:* We examined the CPS for inpatients at the Bundeswehr Hospital Hamburg. With about 300 beds, the Bundeswehr Hospital Hamburg is a medium-size standard healthcare provider. Both military (10%) and civilian patients (90%) are treated in 16 specialist departments. There are also 14 outpatient clinics. With the exception of pediatric and gynecological care, all areas of medical care are offered. The hospital has no pharmaceutical admission department. At the time of our investigation, neither a Computerized Physician Order Entry (CPOE) system nor a Clinical Decision Support System (CDSS) had been implemented. The clinical pharmacy sub-unit is part of the hospital pharmacy.

Only CPS administered to hospitalized patients during their inpatient treatment were evaluated. No prospective follow-up was performed in the outpatient sector. Available information on the implementation of the CPS was retrospectively evaluated. CPS are offered to the medical departments to varying degrees. In cardiology, for example, regular accompaniment by pharmacists during rounds was established. In other areas, e.g., emergency medicine, no pharmacist was on site. Psychiatry is also supplied logistically through a unit dose drug distribution (for drugs that can be placed in plastic bags). A special request included a regular medication analysis of all patients. In addition, consultation requests for specific patients are possible. General, not-patient-tailored activities of the sub-unit, such as education for physicians and nurses and participation in commissions, were excluded from this investigation. The switch of outpatient medication to the in-house formulary without further counseling was not recorded as CPS in this context. What is more, activities in collaboration with other sub-units, such as therapeutic drug monitoring and individual cytostatic compounding, were not part of this investigation. The pharmacy also supplied medications to external facilities outside the hospital, although no CPS provided to those facilities were evaluated in this survey. All other CPS offered by the local hospital pharmacy for all medical departments were included in this investigation. The CPS were provided by 7 well-experienced pharmacists (specialist pharmacist for clinical pharmacy, in part with further specialization or advanced training in this field) and 2 pharmacy interns (under the supervision of the pharmacists). To perform the CPS, in addition to the original literature, the following drug information databases were used for research: *AiDKlinik*^®^ (*Dosing* GmbH, Heidelberg, Germany), *ClinicalKey*^®^ (*Elsevier* GmbH München, Germany), *IDMedics*^®^ (*ID Information und Dokumentation im Gesundheitswesen* GmbH & Co. KGaA, Berlin, Germany), *Lauer-Taxe*^®^ (*Lauer Fischer* GmbH, Fürth, Germany), *Med-IQ*^®^ (*Med-IQ* Baltimore, MD, USA).

*Study design:* Monocentric and retrospective data analysis from the hospital information system and the pharmacy documentation, as well as interviews with pharmacists for evaluation purposes.

*Study protocol:* Following positive ethical approval, we evaluated the patient chart data and the pharmacy documentation on CPS from 9 December 2024 to 31 March 2025. We interviewed the pharmacists who performed the respective CPS about the clinical risks of the DRP they identified in the CPS.

We developed the following instruments for standardization and pre-tested them in advance before use for suitability in an internal expert panel: (a) A Standard Operating Procedure (SOP) for the practical exercise and documentation of CPS by the pharmacists performing them, (b) a standardized form (checklist) for retrospective data collection as part of this study, and (c) a standardized questionnaire for conducting the pharmacist interviews including a risk assessment according to the NCC-MERP score [12], which evaluated quantitatively and differentiated between severity and probability of occurrence. The actual/potential severity has to be assessed in the following categories: very severe, severe, moderate, low, very low, or without impact. The probability of occurrence of DRP has to be assessed in the following categories: very low, low, moderate, high, very high, or actually occurred. In cases of uncertainty regarding classification, a decision was made based on collegial consultation involving at least three clinical pharmacists.

By those methods, we assessed the following topics [data source]:

i. CPS per patient/treatment day [patient chart and pharmacy documentation]
ii. Number of patients involved in CPS per medical department [patient chart and pharmacy documentation]
iii. Receipt of the request for the CPS [pharmacy documentation]
iv. Topic of the CPS [pharmacy documentation]
v. Drug group addressed in CPS [pharmacy documentation]
vi. Time required for CPS [pharmacist interview]
vii. Potential severity of DRP and actually occurring ADR identified during CPS [pharmacist interview]
viii. Probability of occurrence of DRP identified during CPS [pharmacist interview]
ix. Recommendations to solve DRP identified during CPS [pharmacy documentation and pharmacist interview]
x. Acceptance of recommendations to solve DRP identified during CPS [pharmacy documentation and pharmacist interview]

*Data acquisition and analysis:* A pharmacist trainee (W.S.) carried out the data collection, pharmacist interviews, and data analysis. A standardized form (checklist) was created in advance for data collection and pre-tested by the author team (as an expert panel). The pharmacist interview followed a standardized questionnaire including a risk assessment according to the NCC-MERP score [12]. This questionnaire was also agreed upon and pre-tested in advance by the expert panel. The pharmacist trainee (W.S.) was trained in quality assurance for data collection and evaluation before data collection and interviews were started. The data was summarized anonymously using Microsoft Excel (for Windows 11) and evaluated descriptively with regard to the frequency of the questions. Absolute and relative frequencies are given, as appropriate.

## 3. Results

### 3.1. CPS per Patient/Treatment Day

In total, 1000 CPS were documented in 504 patients (mean age: 69.95 years; 229 female, 258 male, and 17 unknown) on 16,705 treatment days. A total of six recommendations per 100 treatment days and two recommendations per patient were forwarded to the treating physicians.

### 3.2. Number of Patients Involved in CPS per Medical Department

**Figure 1:**
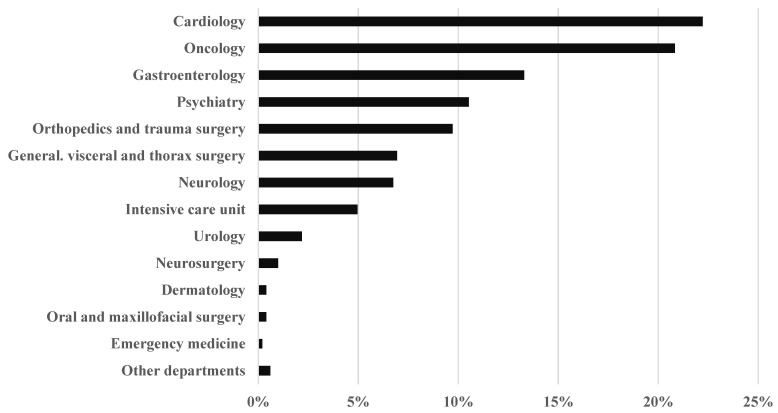
Number of patients involved in CPS per medical department. Total: *n* = 504 patients.

The number of patients per medical department ranged from 105 (20.83%) in oncology and 112 (22.22%) in cardiology to 2 (0.40%) each in oral and maxillofacial surgery and dermatology (Figure 1, Table S1 in Supplementary Materials).

### 3.3. Receipt of the Request for the CPS

Of all CPS, 4.37% were requested verbally/by phone. In writing, 15.48% were requested, e.g., as a consultation. A total of 66.87% were recorded by the pharmacist on ward rounds. As part of a special request, 6.55% came up, 5.56% during medication reviews in the context of unit dose drug distribution, and 1.19% in other contexts.

### 3.4. Topic of the CPS

**Figure 2:**
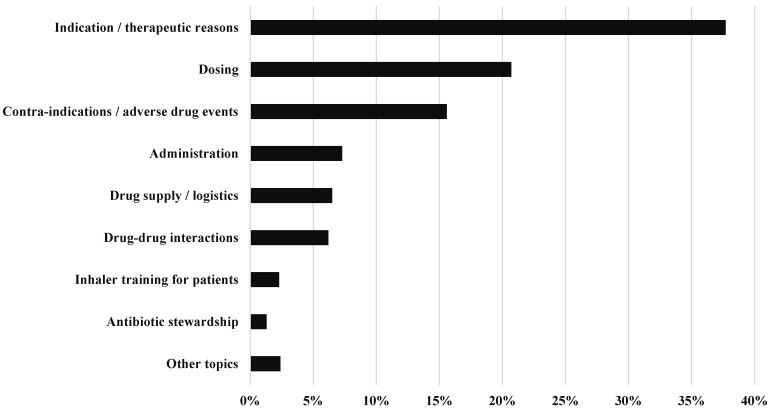
Topic of the CPS. Indication/therapeutic reasons covered all aspects describing a lack of medication in the case of a medical diagnosis or, conversely, medication without a medical diagnosis.

Indications/therapeutic reason was addressed in 37.70% of the CPS, dosing in 20.70%, contraindications/adverse drug reactions in 15.60%, administrations in 7.30%, drug supply/logistics in 6.50%, drug–drug interactions in 6.20%, inhaler training for patients in 2.30%, antibiotic stewardship in 1.30%, and other topics in 2.40% (Figure 2, Table S2 in Supplementary Materials).

### 3.5. Drug Group Addressed in CPS

The 10 drug groups most frequently involved in CPS were as follows: agents for obstructive respiratory diseases R03 (*n* = 126), psycholeptics N05 (*n* = 89), antithrombotic agents B01 (*n* = 87), psychoanaleptics N06 (*n* = 72), agents affecting lipid metabolism C10 (*n* = 66), antidiabetics A10 (*n* = 60), antibiotics for systemic use J01 (*n* = 58), diuretics C03 (*n* = 50), agents affecting the RAAS system C09 (*n* = 46), and analgesics N02 (*n* = 45).

### 3.6. Time Required for CPS

**Figure 3:**
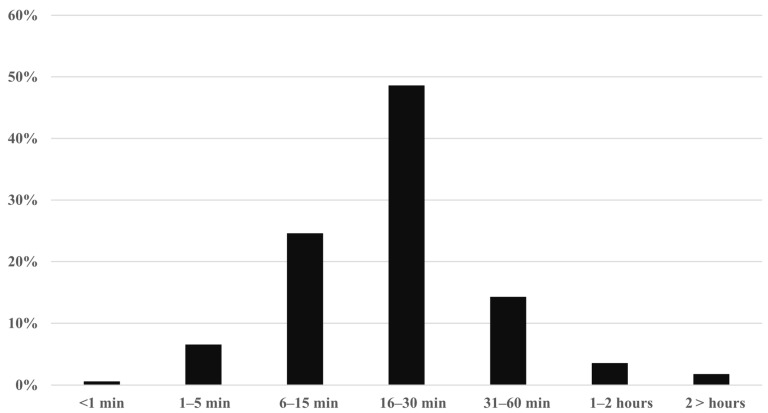
Time required for CPS.

The processing time per CPS was less than 1 min in 0.60%, 1–5 min in 6.55%, 6–15 min in 24.60%, 16–30 min in 48.61%, 31–60 min in 14.29%, 1–2 h or more in 3.57% and more than 2 h in 1.79% (Figure 3, Table S3 in Supplementary Materials).

### 3.7. Potential Severity of DRP and Actually Occurring ADR Identified During CPS

The severity of the identified DRP (percent of *n* = 358 identified DRP) was classified as very severe (5.03%), severe (42.74%), moderate (34.36%), low (15.08%), very low (1.40%), or without impact (1.40%). The severity of actually occurring events predicted by the identified DRP (i.e., ADR, percent of *n* = 55) was classified as very severe (7.27%), severe (30.91%), or moderate (47.27%).

### 3.8. Recommendations to Solve DRP Identified During CPS

In total, 932 recommendations were forwarded to solve DRP identified during CPS.

### 3.9. Acceptance of Recommendations to Solve DRP Identified During CPS

**Figure 4:**
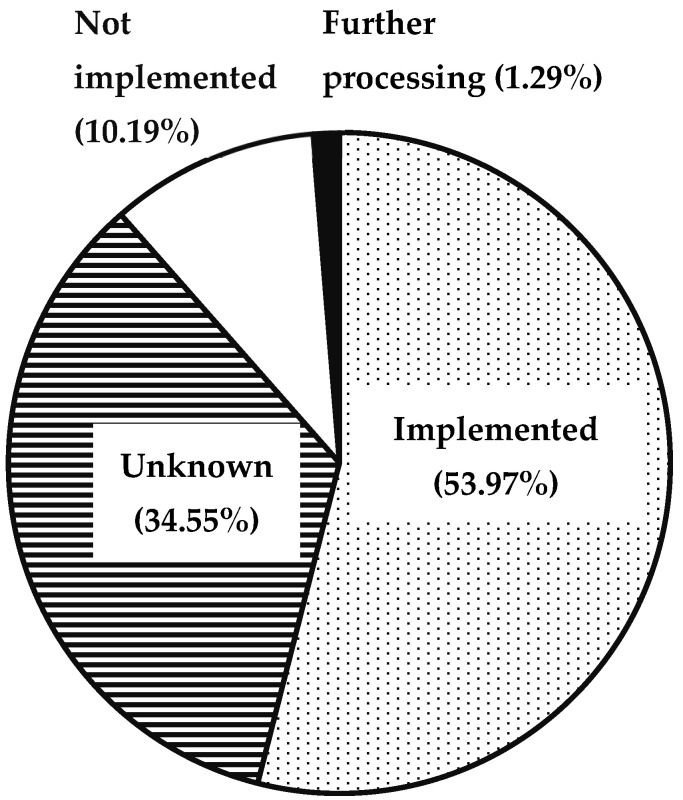
Acceptance of recommendations to solve DRP identified during CPS. Further processing was to the extent that this was recorded in the retrospective data analysis. No prospective follow-up examination was performed to clarify any outstanding processing issues.

From all 932 recommendations, 53.97% were directly implemented on the ward by the treating physicians. Of the recommendations, 1.29% had to be followed up during further treatment. A total of 10.19% were not implemented, and for 34.55%, the implementation was not known or could not be determined (Figure 4, Table S4 in Supplementary Materials).

## 4. Discussion

### 4.1. General Considerations

CPS have the potential to prevent DRP and the resulting ADR. However, in order to be effective and efficient under routine conditions with limited resources, they should be prioritized according to the need and tailored to the medical requirements. As a consequence, available resources should be used optimally in order to increase patient safety as demonstrably as possible on the basis of patient- and quality-based parameters. Therefore, it is important not only scientifically but also for practical implementation and sustainable supply to take stock of which CPS are offered depending on the medical departments. The thematic objectives and the time required should also be considered. Particular attention should be paid to the DRP that are identified and should be resolved. Ultimately, a CPS is only effective if the resulting recommendations are also implemented. It is particularly important to determine whether ADR actually occurred and could be resolved through the recommendations derived from a CPS, or at least what potential was available for this purpose.

### 4.2. Main Findings of This Investigation

The number of 1000 examined CPS in over 500 patients on almost 17,000 treatment days provides an unusually comprehensive picture of CPS in a non-university hospital. The focus was on CPS for inpatients during their hospital stay. The results demonstrate a broad spectrum of CPS offered in a wide variety of medical disciplines. However, a focal point was identified in the areas of oncology, cardiology, and nephrology. It is striking that active participation of pharmacists in the rounds was clearly worthwhile in order to specifically identify and resolve DRP. Only a portion of the questions were actively brought to the pharmacy. It is also striking that the time required for the rounds takes up the most time in absolute terms. One could also say that there are areas such as oncology where CPS are particularly time-consuming. On the other hand, this effort is likely to be effective. In 1000 CPS offered, 932 recommendations were forwarded to resolve DRP. This shows that pharmacists actually offered concrete solution strategies while offering CPS. Of these, more than every second recommendation was implemented by the treating physicians. It should be noted that the number of unfollowed consequences of DRP was high, and even in many of these cases, a positive effect of the CPS can be assumed. The high risk of many of the DRP identified is worrying. In fact, more than 15% of the identified DRP had already resulted in an ADR before any recommendations were given. Even if these were not always serious, it is clear that pharmacists’ recommendations are desirable at an early side of prevention. At the same time, pharmacists should not neglect to offer CPS in a prioritized and economically efficient manner. After all, patient safety in hospitals and the entire healthcare system can only be improved if available resources are used optimally.

### 4.3. Setting

Our hospital has a maximum capacity of 300 beds. However, unlike hospitals of this size, such as those run by municipal or private organizations, the primary focus of a Bundeswehr hospital is the training of healthcare and nursing professionals for tasks within the Bundeswehr. A university hospital also has a research and teaching mandate alongside patient care. In this respect, the characteristics of the Bundeswehr hospital perhaps place it somewhere between smaller private/municipal hospitals and university hospitals. However, the smaller number of beds also means that the pharmacy staff (despite the education mandate) are fewer than at a university hospital. As a result, specialization is not possible in the same way in our hospital as in the university hospital. If there are staff shortages, for example due to illness, a smaller number of pharmacists in our institution should be able to cover for each other than at universities. This also has advantages as expertise from all pharmaceutical fields is retained better in this way. Staff shortages, however, are more quickly noticeable in a smaller hospital. In university hospitals, funding for clinical pharmacists is often provided through the medical departments. At our hospital, however, they have to be remunerated by the pharmacy itself, or there are (very limited) additional funds for establishing CPS. Whether positive or negative, the different conditions make it clear, in our opinion, that CPS should also be evaluated in smaller hospitals and that data from other settings with different conditions or from abroad should not simply be adopted. In our opinion, an independent study, such as the one we conducted here, is advisable.

### 4.4. Methodological Aspects

The striking aspect of this study is the fact that methodologically objective record analysis and subjective evaluations by well-experienced pharmacists (very familiar with the respective CPS) were combined. Routine data from patient files in the hospital information system were successfully combined with the documentation of CPS in the pharmacy. In this way, a comprehensive picture of the clinical situation was obtained through the combination of different methods. Moreover, the study was conducted in a non-university hospital.

Although, partly due to capacity constraints, we only included a relatively short period in this study, based on a comparison with the number and characteristics over several years, we assume that it was representative. Furthermore, with 1000 examined CPS in over 500 patients on almost 17,000 treatment days, we analyzed a fairly large and, we would argue, therefore representative number of CPS in our setting.

We, last but not least, also see a methodological advantage in the fact that one person (W.S.) collected all the data and interviewed the pharmacists. In this way, individual influences in the evaluation by the pharmacists involved were minimized.

### 4.5. Comparison to the Literature

In [13], the authors also evaluated a remarkably high number of 740 interventions for CPS. In contrast to our analysis, they found antibiotics for systemic use and anti-Parkinson’s drug as notable substance classes. We, however, found agents for obstructive respiratory diseases to be the most prominent class. This suggests that the issue of appropriate administration of medications was more frequently raised by us. This is consistent with prescribing errors, which was cited as one of the main themes in [13]. Indication-related themes, however, also occurred frequently in our study, suggesting that the prescribing level of the medication process was also frequently addressed in our CPS.

A comparison with [14] shows that the issues addressed by the CPS are quite similar in our analysis. The authors in [14] reported that medication errors caught by pharmacists include overdoses, underdoses, missed doses, gaps in medication history, allergies, and near issues. Others [15] showed that interventions related to prescribing and overlap errors were highest at 30% and 29%, respectively.

In our analysis, implementation of the CPS recommendations in clinical practice was comparatively low at only around 50%. At first glance, this is in contrast to recent publications that deal with the acceptance of CPS. In [13] for instance, a rate of 93% of interventions being fully accepted by physicians is reported. Although the rate reported by other authors [16] was lower, with an overall acceptance rate of proposed interventions by physicians of 76%, our rate requires explanation. One explanation could be that we evaluated routine care CPS—rather than as part of an explicit planned study where greater awareness and additional factors such as a Hawthorne [17] and Rosenthal effect [18] play a role. Furthermore, we did not limit ourselves to one medical department—perhaps with high potential to evaluate CPS, such as intensive care. We rather included the whole spectrum of medical departments also including perhaps “less worthwhile” areas in regard to CPS. What is more, when comparing implementation rates internationally, the following aspects should also be considered: first, we collected the rates shown here based on routine documentation and not as part of a prospective intervention study. This also explains the high rate (34.55%) of such CPS whose implementation could not be clearly determined. Second, the role of clinical pharmacists in Germany differs considerably from other countries. While in the latter, the pharmacist is often located directly on the ward, in Germany (and also in our setting) they increasingly operate from the pharmacy department. This makes it more difficult to assess the success of CPS on the ward in a follow-up. Third, patient transfers and discharges make follow-up more difficult in German settings due to the stricter intersectoral interfaces between inpatient and outpatient care. Fourth, physicians are not “forced” to justify to pharmacists whether and why recommendations have not been followed. In other countries, pharmaceutical recommendations may be more binding than is legally and factually the case in Germany. A final and particularly important point is that an implementation rate is, particularly in some CPS such as ward round participation, difficult to assess since the final determination of the procedure emerges from an interprofessional discussion (which is also desirable). In this respect, a clear indication of acceptance or rejection of pharmaceutical suggestions is frequently not easy to perform. Nevertheless, a high, albeit not uncritical, acceptance of pharmaceutical suggestions indicates a particular level of trust in the pharmacists’ expertise.

Although there are only few assessments of clinical risk, the existing evaluations provide a good means of comparison to our data: As reported in [13], 63% of interventions were rated as being of moderate and 23% of high clinical importance. We found about one-third of the DRP addressed by CPS to be (very) highly relevant. In contrast to [13], where an assessment was performed by an external panel, we used an assessment by the pharmacists themselves performing the CPS. Our method may be less objective. However, it offers the possibility that the person who can best assess the individual situation was approached.

While the most common interventions in [13] were training of a healthcare professional (20%) and dose modification (16%), our recommendations assessed in this investigation were frequently made in the form of individual assessments during ward rounds.

Compared to other studies [16] with a considerably high DRP rate of about 85%, our rate was low as we found DRP in only half of the individual CPS measures. Authors in [16] reported that 538 DRP were found in 1,724 prescriptions. However, these numbers suggest that we had a different CPS strategy. In our study, a large number of measures were addressed during attendance at the visit and not just during the review of prescriptions.

According to [19], it also makes sense to be particularly involved on the ward. This publication [19] found that the on-ward validation group tended to have a higher acceptance rate compared to a central care group.

We further suspect that we consistently included all individual CPS—even if they did not lead to recommendations. This is—in our experience—lived practice and shows that regular attendance by clinical pharmacists has positive long-term effects, that many situations could be resolved on admission before they could lead to a DRP through ward rounds.

In [14], education initiated by clinical pharmacists led to a better understanding of medication and improved adherence to therapy, higher patient satisfaction, and better control of chronic diseases. This suggests that other authors have also evaluated more long-term approaches. Our focus was on very specific patient-related questions on ward rounds and specific patient-related queries in consultations. Although training courses are also offered at our hospital, they were not evaluated within the analysis presented here.

As recently presented in [20], a comparison of two groups, one for which a CPS concept was offered and another for which it was not, is the best way to show the benefits in principle and to prove concrete effects above and beyond those already achieved by the physicians alone. In the context of our analysis shown here, however, we were concerned with recording the status quo. This was due to the fact that, in principle, CPS has been offered universally in our setting for all medical areas for a considerable time.

An interesting approach that we have not yet investigated is presented in [21]: here, the patients’ perspective was also involved. An ambitious approach for future studies was shown by authors in [22]: A multivariate analysis showed that the involvement of a clinical pharmacist correlated with a shorter length of stay. As described in [23], the experts agreed on other specific outcomes that could also be investigated in future investigations: hospital-acquired complications, readmission due to medication errors, and unplanned readmission within 10 days. Ultimately, the results of evaluations should lead to CPS being better adapted to the needs of users and prioritized [24,25], especially in an environment with limited resources.

New concepts such as centralized, remote, clinical pharmacy [26] could help to make efficient use of scarce clinical pharmacy resources.

### 4.6. Future Perspectives

Based on the results presented here, further developments are planned in the context to better connect CPS at admission and at discharge. While CPS during hospitalization have already been well established in our house, CPS at discharge remains to be expanded. By this, a more sustainable implementation of therapeutic modifications recommended by clinical pharmacists should be reached.

Another area that is to be expanded concerns patient-related counseling. Particularly, inhaler training for patients is already well-accepted and frequently performed. We see, however, a high potential to increase directly patient-related services in the future when the patients are responsible for medication administration themselves.

Last but not least, the assessment of clinical risks, as shown here, as an evaluation by the performing clinical pharmacists should be further developed by assessing clinical outcomes and validated indicators that point out a risk early on before a patient is harmed.

We consider the implementation rate to be a fundamental issue that we want to optimize in the future. Over 50% of our recommendations were implemented. What is more, we can assume that a considerable number of the over 30% of DRP that could not be pursued further have nevertheless been solved as a result of our recommendations. Other recommendations, however, remained unimplemented. This may be for good medical reasons, but it also might require that communication processes should be further improved in the future.

### 4.7. Limitations and Sources of Potential Bias and Confounders

This study has some limitations: First, only one setting was analyzed. Therefore, generalizations to other settings should be made with caution. Second, the analysis covers a current but limited time window. The results are therefore not fully extrapolatable into the future. Third, as the interview of the pharmacists involved could only be carried out after the CPS had been performed, a certain recall bias cannot be ruled out. However, our analysis also allows a better assessment of the clinical consequences than at the time of implementation of the CPS. Fourth, when evaluating this study, it should be considered that we based our analysis only on the requested CPS. We did not collect all DRP/ADR in our setting and therefore cannot meaningfully determine the fraction of DRP/ADR in our setting for which CPS were offered. We instead focused on CPS, including its clinical implementation. Additionally, the DRP/ADR underlying the CPS were evaluated for their clinical relevance within this study. Fifth, when interpreting the frequency of CPS per medical department, it should be noted that the number of CPS also varies between departments due to differences in the services offered within the CPS. For example, regular visits by pharmacists were implemented in cardiology, whereas in emergency medicine, there was no pharmacist on site.

## 5. Conclusions

In this study, a respectable number of 1000 CPS in 504 patients on 16,705 treatment days were examined. The pharmacists’ participation in the ward rounds induced two-thirds of the CPS. “Indications” was the most frequently addressed topic. “Active substances for obstructive airway diseases” was the most frequently addressed drug group. Oncology, cardiology, and nephrology took the most time of the performed CPS. In oncology, most individual measures were carried out regarding CPS. In 1000 CPS interventions, 932 recommendations were forwarded to the treating physicians to solve the DRP identified during the CPS. Of these recommendations, more than half were directly implemented. Almost a third of the DRP, in which recommendations were made, were categorized as at least moderately severe. In about 15% of identified DRP, an ADR actually had occurred, indicating the need for CPS in future care to improve patient safety.

## Data Availability

The datasets presented in this article are not readily available because the data are part of an ongoing study. Requests to access the datasets should be directed to the corresponding author.

## Supplementary Materials

The following supporting information can be downloaded at: https://www.mdpi.com/article/10.3390/pharmacy13060164/s1, Table S1: Number of patients involved in CPS per medical department; Table S2: Topic of the CPS; Table S3: Time required for CPS; Table S4: Acceptance of recommendations to solve DRP identified during CPS.

## List of Abbreviations

**Abbreviation**	**Full Term**
ADR	Adverse Drug Reaction
CPS	Clinical Pharmacy Services
DRP	Drug-Related Problem
SOP	Standard Operating Procedure
NCC-MERP	National Coordinating Council for Medication Error Reporting and Prevention
CPOE	Computerized Physician Order Entry
CDSS	Clinical Decision Support System
