# Supplementary material for: The Spectrum of Clinical Pharmacy Services in a Non-University Hospital—A Comprehensive Characterization Including a Risk Assessment for Drug-Related Problems and Adverse Drug Reactions
Source: Pharmacy (Basel). 2025 Nov 6;13(6):164. doi: 10.3390/pharmacy13060164 (PMC12641981; doi:10.3390/pharmacy13060164)
Supplement: Supplementary file 1 [file pharmacy-13-00164-s001.zip › pharmacy-3938596-supplementary.pdf]

Table S1. Number of patients involved in CPS per medical department [number relative to total patients with CPS/total number of CPS]. *): for the total department of internal medicine; -: not assessed. CPS: Clinical Pharmacy Services.

Medical department	Patients	CPS	Patient days	Interventions on 100 patient days	Average number of interventions per patient
**Total**	504 [100%]	1000 [100%]	16705	5.99	1.98
**Intensive care unit**	25 [4.96%]	54 [5.40%]	899	6.01	2.16
**Orthopedics and trauma surgery**	49 [9.72%]	121 [12.10%]	1828	6.62	2.47
**Dermatology**	2 [0.40%]	2 [0.20%]	550	0.36	1.0
**General. visceral and thorax surgery**	35 [6.94%]	89 [8.90%]	1230	7.24	2.54
**Neurology**	34 [6.75%]	79 [7.90%]	825	9.58	2.32
**Oral and maxillofacial surgery**	2 [0.40%]	3 [0.30%]	754	0.40	1.50
**Neurosurgery**	5 [0.99%]	17 [1.70%]	576	2.95	3.40
**Urology**	11 [2.18%]	18 [1.80%]	1430	1.26	1.64
**Oncology**	105 [20.83%]	199 [19.90%]	4266*)	12.47*)	1.90
**Cardiology**	112 [22.22%]	201 [20.10%]	4266*)	12.47*)	1.79
**Gastroenterology**	67 [13.29%]	132 [13.20%]	4266*)	12.47*)	1.97
**Psychiatry**	53 [10.52%]	80 [8.0%]	2071	3.86	1.51
**Emergency medicine**	1 [0.20%]	1 [0.10%]	1	100.0	1.0
**Other departments**	3 [0.60%]	4 [0.40%]	-	-	1.33

Table S2. Topic of the CPS [number relative to the number of CPS in total].

Medical department	Dosing	Admin-istration	Drug-drug inter-action	Indication / therapeutic reason	Contra-indications /adverse drug event	Drug supply / logistics	Inhaler training for patientss	Antibiotic stewardship	Other topics
**Total**	207 [20.70%]	73 [7.30%]	62 [6.20%]	377 [37.70%]	156 [15.60%]	65 [6.50%]	23 [2.30%]	13 [1.30%]	24 [2.40%]
**Intensive care unit**	7 [0.70%]	16 [1.60%]	3 [0.30%]	16 [1.60%]	3 [0.30%]	4 [0.40%]	0 [0%]	3 [0.30%]	2 [0.20%]
**Orthopedics and trauma surgery**	23 [2.30%]	7 [0.70%]	11 [1.10%]	60 [6.0%]	12 [1.20%]	5 [0.50%]	1 [0.10%]	1 [0.10%]	1 [0.10%]
**Dermatology**	1 [0.10%]	0 [0%]	0 [0%]	0 [0%]	0 [0%]	1 [0.10%]	0 [0%]	0 [0%]	0 [0%]
**General. visceral and thorax surgery**	12 [1.20%]	11 [1.10%]	7 [0.70%]	37 [3.70%]	14 [1.40%]	5 [0.50%]	0 [0%]	3 [0.30%]	0 [0%]
**Neurology**	19 [1.90%]	2 [0.20%]	6 [0.60%]	29 [2.90%]	19 [1.90%]	2 [0.20%]	0 [0%]	0 [0%]	2 [0.20%]
**Oral and maxillofacial surgery**	1 [0.10%]	1 [0.10%]	0 [0%]	0 [0%]	0 [0%]	0 [0%]	0 [0%]	1 [0.10%]	0 [0%]
**Neurosurgery**	0 [0%]	3 [0.30%]	3 [0.30%]	5 [0.50%]	4 [0.40%]	1 [0.10%]	0 [0%]	0 [0%]	1 [0.10%]
**Urology**	4 [0.40%]	2 [0.20%]	0 [0%]	3 [0.30%]	1 [0.10%]	4 [0.40%]	0 [0%]	3 [0.30%]	1 [0.10%]
**Oncology**	35 [3.50%]	10 [1.0%]	9 [0.90%]	86 [8.60%]	31 [3.10%]	6 [0.60%]	11 [1.10%]	1 [0.10%]	10 [1.0%]
**Cardiology**	57 [5.70%]	7 [0.70%]	7 [0.70%]	79 [7.90%]	32 [3.20%]	7 [0.70%]	9 [0.90%]	1 [0.10%]	2 [0.20%]
**Gastroenterology**	28 [2.80%]	5 [0.50%]	8 [0.80%]	50 [5.0%]	24 [2.40%]	10 [1.0%]	2 [0.20%]	0 [0%]	5 [0.50%]
**Psychiatry**	19 [1.90%]	9 [0.90%]	7 [0.70%]	10 [1.0%]	16 [1.60%]	19 [1.90%]	0 [0%]	0 [0%]	0 [0%]
**Emergency medicine**	0 [0%]	0 [0%]	0 [0%]	1 [0.10%]	0 [0%]	0 [0%]	0 [0%]	0 [0%]	0 [0%]
**Other departments**	1 [0.10%]	0 [0%]	1 [0.10%]	1 [0.10%]	0 [0%]	1 [0.10%]	0 [0%]	0 [0%]	0 [0%]

Table S3. Time required for CPS.

Medical department	<1 min	1-5 min	6-15 min	16-30 min	31-60 min	1-2 hours	>2 hours
**Total**	3 [0.60%]	33 [6.55%]	124 [24.60%]	245 [48.61%]	72 [14.29%]	18 [3.57%]	9 [1.79%]
**Intensive care unit**	0 [0%]	2 [0.40%]	10 [1.98%]	11 [2.18%]	1 [0.20%]	0 [0%]	1 [0.20%]
**Orthopedics and trauma surgery**	0 [0%]	10 [1.98%]	11 [2.18%]	16 [3.17%]	6 [1.19%]	3 [0.60%]	3 [0.60%]
**Dermatology**	0 [0%]	1 [0.20%]	1 [0.20%]	0 [0%]	0 [0%]	0 [0%]	0 [0%]
**General. visceral and thorax surgery**	0 [0%]	2 [0.40%]	20 [3.97%]	4 [0.79%]	4 [0.79%]	4 [0.79%]	1 [0.20%]
**Neurology**	0 [0%]	2 [0.40%]	17 [3.37%]	6 [1.19%]	6 [1.19%]	3 [0.60%]	0 [0%]
**Oral and maxillofacial surgery**	0 [0%]	0 [0%]	2 [0.40%]	0 [0%]	0 [0%]	0 [0%]	0 [0%]
**Neurosurgery**	0 [0%]	1 [0.20%]	2 [0.40%]	0 [0%]	1 [0.20%]	0 [0%]	1 [0.20%]
**Urology**	0 [0%]	4 [0.79%]	6 [1.19%]	1 [0.20%]	0 [0%]	0 [0%]	0 [0%]
**Oncology**	1 [0.20%]	1 [0.20%]	3 [0.60%]	78 [15.48%]	21 [4.17%]	1 [0.20%]	0 [0%]
**Cardiology**	0 [0%]	1 [0.20%]	4 [0.79%]	89 [17.66%]	16 [3.17%]	1 [0.20%]	1 [0.20%]
**Gastroenterology**	0 [0%]	1 [0.20%]	20 [3.97%]	32 [6.35%]	12 [2.38%]	2 [0.40%]	0 [0%]
**Psychiatry**	2 [0.40%]	7 [1.39%]	27 [5.36%]	7 [1.39%]	5 [0.99%]	3 [0.60%]	2 [0.40%]
**Emergency medicine**	0 [0%]	0 [0%]	0 [0%]	0 [0%]	0 [0%]	1 [0.20%]	0 [0%]
**Other departments**	0 [0%]	1 [0.20%]	1 [0.20%]	1 [0.20%]	0 [0%]	0 [0%]	0 [0%]

Table S4. Acceptance of recommendations to solve DRP identified during CPS.

Medical department	Further processing	Implemented	Not implemented	Unknown
**Total**	12<br>[1.29%]	503<br>[53.97%]	95<br>[10.19%]	322<br>[34.55%]
**Intensive care unit**	1<br>[0.11%]	18<br>[1.93%]	0<br>[0%]	27<br>[2.90%]
**Orthopedics and trauma surgery**	0<br>[0%]	41<br>[4.40%]	27<br>[2.90%]	48<br>[5.15%]
**Dermatology**	0<br>[0%]	2<br>[0.21%]	0<br>[0%]	0<br>[0%]
**General. visceral and thorax surgery**	0<br>[0%]	42<br>[4.51%]	7<br>[0.75%]	36<br>[3.86%]
**Neurology**	0<br>[0%]	45<br>[4.83%]	4<br>[0.43%]	22<br>[2.36%]
**Oral and maxillofacial surgery**	0<br>[0%]	2<br>[0.21%]	0<br>[0%]	0<br>[0%]
**Neurosurgery**	0<br>[0%]	3<br>[0.32%]	0<br>[0%]	10<br>[1.07%]
**Urology**	0<br>[0%]	10<br>[1.07%]	0<br>[0%]	2<br>[0.21%]
**Oncology**	3<br>[0.32%]	103<br>[11.05%]	26<br>[2.79%]	55<br>[5.90%]
**Cardiology**	4<br>[0.43%]	122<br>[13.09%]	16<br>[1.72%]	45<br>[4.83%]
**Gastroenterology**	4<br>[0.43%]	74<br>[7.94%]	9<br>[0.97%]	51<br>[5.47%]
**Psychiatry**	0<br>[0%]	41<br>[4.40%]	6<br>[0.64%]	22<br>[2.36%]
**Emergency medicine**	0<br>[0%]	0<br>[0%]	0<br>[0%]	1<br>[0.11%]
**Other departments**	0<br>[0%]	0<br>[0%]	0<br>[0%]	3<br>[0.32%]
